# Flow Cytometric Evaluation of Cell Cycle Regulators (Cyclins and Cyclin-Dependent Kinase Inhibitors) Expressed on Bone Marrow Cells in Patients with Chronic Myeloid Leukemia and Multiple Myeloma

**DOI:** 10.5505/tjh.2012.33602

**Published:** 2011-04-28

**Authors:** Selami K Toprak, Klara Dalva, Merih Kızıl Çakar, Nazmiye Kurşun, Meral Beksaç

**Affiliations:** 1 Ankara University, School of Medicine Department of Hematology, Ankara, Turkey; 2 Ankara University, School of Medicine Department of Biostatistics, Ankara, Turkey

**Keywords:** Chronic myeloid leukemia, Cyclin, Cyclin dependent kinase inhibitor, Flow cytometry, Multiple myeloma

## Abstract

**Objective:** The aim of this study was to use flow cytometry to analyze the expression of cell cycle-regulating elementswith low and high proliferative signatures in patients with malignant diseases.

**Material and Methods:** Cyclin D, E, A, and B, and cyclin-dependent kinase inhibitor (CDKI) p16 and p21 levels weremeasured via flow cytometry in patients with chronic myeloid leukemia (CML) (n = 16) and multiple myeloma (MM)(n = 13), and in controls (n = 15).

**Results:** The distributions of the cell cycle S phase were 10, 63%, 6, 72% and 3, 59%; for CML, MM and controlpatients, respectively. Among all the cyclins expressed during the S phase, cyclin D expression was the lowest in the CMLpatients. Distribution of cyclins and CDKIs during the G2/M phase was similar in the MM and control groups, whereascyclin expression was similar during all 3 phases in the MM and CML groups.

**Conclusion:** Elevated cyclin expression during cell cycle phases in the CML and MM patients was not associatedwith elevated CDKI expression. This finding may increase our understanding of the mechanisms involved in theetiopathogenesis of hematological malignancy.

## Abstract (tr)

**Amaç:** Bu çalışmanın amacı, yüksek ve düşük proliferasyon hızına sahip malign hastalıklarda, hücre siklusunu düzenleyen faktörlerin flow sitometrik olarak analiz edilmesidir.

**Gereç ve Yöntemler:** Kronik miyelositer lösemi (KML) (n=16), Multipl Miyelom (MM) (n=13) ve kontrol (n=15) olgularında siklin D, E, A, B ve siklin bağımlı kinaz inhibitörü (SBKİ) p16, p21 düzeyleri akımsitometrik yöntemle ölçülmüş ve değerlendirilmiştir.

**Bulgular:** KML, MM ve kontrol olgularındaki S evresi dağılımı sırasıyla % 10, 63; % 6, 72 ve % 3, 59 olarak saptanmıştır. KML grubunda siklin D ekspresyonu, S evresindeki diğer siklinlere göre en düşük düzeyde bulunmuştur. G2/M evresinde, MM ve kontrol gruplarındaki siklin ve SBKİ düzeyleri birbirleriyle benzer saptanırken, MM ve KML gruplarının her üç evresindeki siklin ekspresyonları ise paralel bulunmuştur.

**Sonuç:** Kontrol grubuyla karşılaştırıldığında hasta gruplarında, siklin ekspresyonlarının artışına SBKİ düzeylerinin artışının eşlik etmediği saptanmıştır. Bu bulgu, belki de hematolojik malign hastalıkların etyopatogenezinin açıklanmasına katkıda bulunabilecektir.

## INTRODUCTION

Tissue homeostasis is dependent on the perfect balancebetween cell proliferation and cell death [1]. Proliferationof cells occurs following consecutive events and stages.Dysregulated cell cycle control is a fundamental characteristicof cancers [2]. Normal cells only proliferate inresponse to developmental or other mitogenic signals thatindicate a requirement for tissue growth, whereas the proliferationof cancer cells proceeds essentially unchecked[2]. An understanding of the molecular details of cell cycleregulation and checkpoint abnormalities in cancer, andhow these control mechanisms can be manipulated couldprovide insight into potential therapeutic strategies [3].

The cell division cycle is regulated by fluctuation incyclin-dependent kinase (CDK) and cyclin pairs activity[4]. CDK activity requires binding to regulatory subunitsknown as cyclins [5]. CDK-cyclin complexes include 3interphase CDKs (CDK2, CDK4, and CDK6), a mitoticCDK (CDK1, also known as cell division control protein2), and 4 different classes of cyclins (A-, B-, D-, and E-typecyclins) [5]. The transition of cells through the earlyG1 stage of the cell cycle is coordinated by the activityof CDK4 and CDK6 complexes that are formed followingthe mitogen-dependent expression of D-type cyclins(D1, D2, and D3) [6]. CDK4/6-type-D cyclin complexesphosphorylate and inactivate retinoblastoma family protein(pRb), resulting in the release of E2F transcriptionfactors that control the expression of the genes requiredfor G1/synthesis phase transition and synthesis to S phaseprogression [4]. Inactivation of pRb facilitates expressionof E-type cyclins that bind and activate CDK2 during thelate G1 and early S phases. In reference to such studies,CDK2-cyclin A were implicated in committing a cell to thecompletion of S phase [7].

Despite requiring phosphorylation, CDK-cyclin complexesare kept inactivated by binding to a CDK inhibitor(CDKI). CDK activity is regulated by 2 families of inhibitors:INK4 proteins, including INK4A (p16), INK4B (p15),INK4C (p18), and INK4D (p19), and the Cip and Kipfamily, which is composed of p21 (Cip1), p27 (Kip1), andp57 (Kip2) [5,8]. In general, when the INK group functionsin the genetic pathway containing cyclin D-CDK4/6-pRb and E2F, the Cip/Kip group can inhibit CDK2 kinaseand CDK4/6 [9,10].

Recent research indicates that CDK down regulationmay result in defective homeostasis in specific tissues andthat CDK hyperactivation may facilitate tumor developmentby inducing unscheduled cell division in stem andprogenitor cells [5]. CDKs are targets for cancer therapy;their expression is often perturbed in cases of malignancyand their inhibition can induce apoptosis [11]. Cellularcheckpoint integrity is often lost as a result of CDKI inactivationor cyclin overexpression [11].

Multiple myeloma (MM) is a malignant neoplasm thatarises from plasma cells of low proliferative potential [12].Translocations involving the immunoglobulin heavy chainregion (IgH) on chromosome 14q32 are an importantcytogenetic event in the pathogenesis of various B-celllymphoid neoplasms such as MM. To date, approximatelymore than 20 different chromosomal partner regions thattranslocate to 14q32 have been identified in MM, of whicht(11;14)(q13;q32) is the most common translocation, witha reported frequency of 15%-20% based on fluorescencein situ hybridization (FISH) and conventional cytogeneticanalysis [13].

Although myeloma tumors exhibit complex karyotypes,and a variety of structural and quantitative chromosomalabnormalities, these tumors are unified in theirubiquitous targeting of cyclin D genes for overexpression[14]. In all, 54% of myeloma tumors overexpress cyclinD1 (CCND1), 48% overexpress cyclin D2 (CCND2), 3%overexpress cyclin D3 (CCND3), and 8% overexpressboth CCND1 and CCND2 [15]. Recently, translocationand cyclin (TC) classification of MM has been introduced;the classification is based on cyclin expression types [16].Thus, CDK inhibitors have a potential role in the treatmentof MM, including PD 0332991, a specific inhibitorof CDK4/6, and seliciclib, UCN-01, P276-00, AT7519,and RGB 286638, non-specific CDK inhibitors [12].

Chronic myeloid leukemia (CML) is a clonal myeloproliferativedisorder characterized by a chromosomaltranslocation (9;22) (q34; q11) that produces the oncogenicBcr-Abl fusion protein, resulting in a constitutivelyactive tyrosine kinase with high proliferative potential[17]. Despite progress in the treatment of early-stage CML,the accelerated and blastic phases of CML remain a therapeuticchallenge; therefore, novel treatment approachesare needed [17]. Few data are available concerning theexpression status of such cell cycle regulators as cyclinsand CDKIs in CML [18]. Recent data show that low orundetectable expression of CDKI genes is a significantmarker for the active phase of the disease; therefore, sev-eral new molecules are now being tested—alone and incombination with imatinib—to overcome accelerated andblastic phases. Indirubin is a CDKI that has been used intraditional medicine for hundreds of years and is currentlybeing used in clinical trials for CML [19].

The aim of the present study was to use flow cytometryto analyze the expression of nuclear cell cycle-regulatingelements in patients with MM and CML, diseaseswith a low and high proliferation signature, respectively.In normal and hematologically malignant cells partial illuminationof the cell cycle—and thus the etiopathology ofmalignancy—can only be determined via comparison ofthe quantified changes in the cyclical phases of cyclins andCDKIs in healthy and malignant proliferated cells.

## MATERIALS AND METHODS

**Patients and their Characteristics**

Following ethics committee approval of the study protocoland obtaining written informed consent from eachpatient, 16 consecutive CML patients, 13 consecutiveMM patients, and 15 controls were included in the study.Mean age of the CML patients (9 male and 7 female) was44.5 ± 8.2 years (rage: 22-59 years), versus 54.5 ± 4.5years (range: 49-66 years) in the MM patients (8 maleand 5 female). Mean age of the controls (7 male and 8female), who regardless of diagnosis had histopathologicallyproven normal bone marrow, was 45.8 ± 11.8 years(range: 26-71 years). Table 1 summarizes the characteristicsof the patients and controls. Prognosis in the CMLpatients was determined according to the Sokal Index andstaging of the MM patients was based on the InternationalStaging System (ISS) (Table 1) [20,21]. Samples that wereobtained for diagnoses were used in this prospective study.

**Flow Cytometry Principle**

We used specific monoclonal antibodies and flowcytometry to determine cellular expression of cyclins(cyclin A, B, D, and E) and cyclin inhibitors (p16 and p21)throughout the cell cycle.

**Preparation of Cells**

Mononuclear cells (MNCs) were isolated from selectedbone marrow (BM) specimens collected in tubes containingethylene diamine tetraacetic acid (EDTA) usinga density gradient (Ficoll 1.077, Biochrom KG, Berlin,Germany). Following isolation of MNCs, a cell count wasperformed before the cells were fixed in a methanol-free1% formaldehyde solution for 15 min at 4 °C. Followinga washing step performed with phosphate-buffered saline(PBS) containing 1% fetal calf serum (FCS, Sigma, St. Lois,USA), fixation was repeated with 80% ethanol while vortexingthe cells in order to prevent the formation of aggregates.The fixed cells were kept in 80% ethanol at –20 °Cuntil the assay was performed. Before the assay, the cellswere washed with wash buffer (WB) at 400 g for 10 min,so as to remove the ethanol, and then incubated with WBcontaining Triton X-100 for 5 min at 4 °C in order to perforatethe cells.

**Monoclonal Antibodies**

We used the FITC conjugates of the antibodies specificto cyclins A, B, and D, and inhibitor p16 (Pharmingen,USA) and their isotopic controls. The antibodies specificto cyclin E and inhibitor p21 were pure and labeled witha secondary antibody conjugated with FITC (GAM-FITCPharmingen, USA) (Table 2). We used isotypic controls inorder to check for unspecific binding and to set the markersduring the evaluation of the results.

**Assay**

Following distribution of 100 mL of cells (106 cellstube—1) in each tube containing specific antibodies or isotypiccontrols, cells were incubated for 16 h at 4 °C. Atthe end of the first incubation secondary antibodies wereadded to the number 7, 8, and 9 tubes, which containedthe pure antibodies, followed by incubation for 1 h at roomtemperature. During this period all other tubes were keptat 4 °C. Following incubation, cells were washed with 2mL of WB (spun at 400 g for 5 min). Upon elimination ofunbound material, the tubes were incubated with 0.5 mLof propidium iodide for 10 min (PI; 10 mg mL–1, Medac,Germany) in order to label cellular DNA.

The tubes were maintained at 4 °C until data acquisition,which was performed within 1 h of the final incubation.

**Cell Acquisition**

Data acquisition was performed using a FACS Caliburflow cytometry instrument (Becton Dickinson San Jose,CA, USA) after daily checks were made using Calibritesand CENs (Chicken Erytocyte Nucleus). We collected andanalyzed the data for each tube using CellQuest software.

We detected and recorded signals that originated fromFSC, SSC, FL1, FL2, FL2Width, and FL2Area. We also performedcell cycle analysis using ModFit software (Verity SoftwareHouse, ME, USA).

**Analysis**

Before cell cycle analysis, aggregated cells were eliminatedusing the FL2Width-FL2Area distribution of cells. Allanalyses were performed in this selected single cell population.Upon the definition of G0/G1, S, and G2/M phasesbased on the FL2Area parameter that expressed the DNAcontent of the cell, the level of expression of cyclins A,B, D, and E, and inhibitors p21 and p16 was evaluatedseparately for each phase. We also analyzed the cell cyclephases using ModFit software, which uses a differentmathematical approach to fit the FL2Area curve.

**Statistics**

Kruskal-Wallis analysis was used for comparison ofcyclin and inhibitor expression levels during each phaseof the cell cycle. When a statistically significant differencewas established between these two patients and one controlgroups, the multiple comparisons test was used toinvestigate the each group.

Apart from this, analysis of cyclin and inhibitor datawas performed within each group separately dependingon changes of each phase using the Wilcoxon signed-ranktest.

## RESULTS

**Cell Cycle Phase Evaluation**

The distribution of the cell cycle phases based on analysisusing ModFit and CellQuest software is shown in Table3. There was a trend towards a higher percentage of cellsin the S phase for the CML group, while the values werenot significantly different (P = 0.536).

DNA analysis of the control samples showed an abundanceof G0/G1 phase cells, as expected; however, theCML and MM patients had more cells in the S phase (Figure),although the number of cells in the S phase was similarlylow in the MM and control groups. As the number ofcontrol cells in the G2/M phase was insufficient, cyclin andCDKI measurements could not be performed in this group.

**Distribution of cell Cycle Regulators in the Control Group According cell Cycle Phases**

Comparison of the cell cycle-regulating elements cyclinA, B, D, and E, and CDKIs p16 and p21 in the controlgroup showed that cyclin D was expressed most frequentlyduring the G0/G1 phase; the other cyclins werepresent in 33% of the cells, and the cyclin E level was verylow. Both CDK inhibitors (p16 and p21) were detectable,though p16 was expressed at a higher level. During theS phase all cyclins, except for cyclin E, were present in66% of the cells. The relationship with p16 and p21 in theS phase was similar to the G0/G1 phase. No cells in theG2/M phase were detected in the control group.

**Cell Cycle Regulators in the Patients**

**CML Patients**

Expression of cyclins A, B, D, and E, and CDKIs p21and p16 during the G0/G1 and S phases were similar inthe CML patients and controls (P > 0.05); however, thiswas due to the similarity in the change observed from theG0/G1 to S phase in the controls. Cyclin D had the lowestlevel of expression of all the cyclins during the S phase,unlike in the control group (P < 0.05).

**MM Patients**

Distribution of cyclins during the G0/G1, S, and G2/Mphases was similar in the MM and CML patients. Exceptfor the G2/M phase, a similar pattern of cyclin and CDKIexpression was observed in the controls and MM patients.Tables 4 and 5 summarize the results of the comparisonsof cyclin and CDKI expression patterns during the cellcycle phases in the 2 patient groups and the control group.

## DISCUSSION

The present study used flow cytometry to analyze theexpression of cyclins A, B, D, and E, and CDKIs p21 andp16 in patients with CML and MM, and controls. Expressionof cyclins D and E is expected in eukaryotes duringthe G1 phase [22]. When passing through the R checkpointcyclin type D is expressed [23]; thus, the expressionof cyclin D as the major cyclin occurs during the G0/G1phase [24]. Comparison of the G0/G1 and S phases in the present study showed that D-type cyclin expression waslower during the S phase (P < 0.01). According to the literature,cyclin E plays a role in the transition of cells fromthe G1 to S phase [25-27].

In the present study cyclin E expression in the controlgroup during G0/G1 and S phases did not differ significantly.Flow cytometry may not be the ideal technique fordifferentiating early and late cell phases; thus, the findingthat cyclin E expression during the S phase was similarto that during the G0/G1 phase might have been becausethe cells that were measured were in the early S phase.Cyclins A and B, which are referred to as mitotic cyclins,are initially produced following the start of the S phase,and then promote the subsequent phases of the cell cycle[8,28,29]. As the expression of cyclin A begins during thelate G1-early S phase, cyclin B expression occurs duringthe late S phase and peaks through the G2 phase [30,31].

It is well known that cyclin A is responsible from thecontinuation of the S phase and DNA replication at thisphase, expression of cyclin B triggers the end of the G2phase and initiation of mitosis [32-34].

In the present study, in accordance with the literature,expression of cyclins during the S phase in the controlgroup was significantly higher than that during the G0/G1 phase (cyclin A: P < 0.01; cyclin B: P < 0.05). As mostof the cells in the control group were resting, the G2/Mphase could not be observed. CDKI p16 expression in thecontrol group was significantly higher during the G0/G1phase than during the S phase (P < 0.05), whereas CDKIp21 did not follow this pattern.

In the present study’s CML group expression of cyclinD, which belongs to the G1 cyclins group, was maximalduring the G0/G1 phase, as expected. Cyclin D expressionduring the S phase was lower than that during the G0/G1phase (P < 0.001). Based on data from the literature, cyclinD has an evident expression during the G0/G1 phase inCML, but can be higher during the later phases of the cellcycle [35-40].

Expression of the other G1 type cyclin in the presentstudy—cyclin E—did not differ from that of cyclin D. Maximalexpression of cyclin E was observed during the G0/G1 phase, which was higher than that during the S phase(P < 0.05). In the present study cyclin E expression duringthe last phase was significantly lower than during the G1phase, which is agreement with Gong et al.’s results [38].Cyclin E expression in the present study’s CML group wascomparable to that reported by Qin et al. [41].

In the present study expression of cyclin A—a mitoticcyclin—was similar in the CML and control groups. CyclinA expression was higher during the S phase than during theG0/G1 phase (P < 0.001), and was higher during the G2/Mphase than during the G0/G1 phase (P < 0.05), which isin agreement with Paterlini et al. [39]. Koeffler et al. comparedthe expression of cyclin A1 in normal and leukemichematopoietic cell lines using RT-PCR and reported thatthis cyclin was overexpressed in the leukemic lines [42].Kramer et al. used RT-PCR and reported that cyclin A1was present in 84 of 113 CML patients [32].

In the present study expression of the other mitoticcyclin—cyclin B—was similar to that of cyclin A throughoutthe cell cycle. Cyclin B expression was significantlyhigher during the S phase than during the G0/G1 phase(P < 0.001), and was higher during the G2/M phase thanduring the G0/G1 phase (P < 0.05). Cyclin B expressionduring the S and G2/M phases did not differ significantly,as expected. Gorczyca et al. [31] reported cyclin B1 overexpressionduring the S phase fraction and in accordancewith the present study although cyclin B1 expression wasnot observed during the S and G2/M phases in lymphocytesadministered phytohemagglutinin to stimulate proliferation,its expression was high during all 3 phases intumoral samples. In the present study cyclin B expressionwas observed during the first phase in the CML patients,which is similar to the results Ma et al. reported in acuteleukemia patients [43].

The expression of p16 in the present study differedbetween the controls and patients; it occurred during boththe G0/G1 and S phases in the control group, but duringall 3 phases in the CML group, and its level of expression in the CML group during the S phase was significantlyhigher than that in the control group (P < 0.05). When thecyclin D levels of the G0/G1 and S phases were examined,there was no statistically significant difference between theCML and control groups.

In addition, while cyclin D expression was significantlyhigher during the G0/G1 phase than during the Sphase in the CML group, expression of its inhibitor (p16)was lower during the G0/G1 phase than during the otherphases, suggesting an imbalance that facilitates leukemicprogression. Hirose et al. did not observe any p16 expressiondespite the fact that cyclin D1 and CDK4 expressionwas observed in 16 of the 17 lines they examined [44].

In the present study p21 was expressed at a similar levelduring all 3 phases in the CML patients; however, moreimportantly cyclin A expression was higher during the Sphase in the control group than in the CML group (P <0.01), whereas p21 expression was similar. This findingsuggests another imbalance between cyclins and CDKIs thatfacilitates malignant cellular proliferation. Cyclin D expressionin MM patients has been studied extensively [45-48];however, findings concerning the phase during which itsexpression is highest are inconclusive. In some studies p16protein was observed in mature cell lines, whereas cyclinD1 was highly expressed in immature cells [49].

Cyclin D1 expression was at its peak during the G0/G1 phase in the present study’s MM group, as expected.Cyclin D1 expression was significantly higher during theG0/G1 phase than during the S phase in the MM group (P< 0.01), and was higher during the G0/G1 phase than duringthe G2/M phase (P < 0.05). Expression of cyclin D1—aG1 cyclin—during the S and G2/M phases did not differsignificantly. A study by Sonoki et al. included 20 patientswith plasma cell malignancies that were analyzed usingthe Northern blot. Cyclin D1 expression was observed in6 of their 17 MM cases and all 3 plasma cell leukemiacases [45]. Pruneri et al. reported that the rate of cyclinD1 overexpression was 25% among 48 MM patients [46].Hoechtlen-Vollmar et al. [50] reported cyclin D1 expressionin 19 of 50 MM cases, which is similar to the presentstudy’s findings. The researchers also reported that beta-2microglobulin and cyclin D1 amplification can be usedtogether to predict duration of survival. Based on the presentstudy’s results and those previously published, cyclinD1 expression is highest during the first phase and graduallydecreases during the subsequent phases.

In the present study’s MM group p16 expressionoccurred at a similar level during all 3 phases of the cellcycle. CDKI p16 expression the in MM group was similar to that in the control group. The level of expressionof p16 did not follow the overexpression of cyclin Dduring the G0/G1 phase, which was another imbalancebetween cyclins and CDKIs. Kawano et al. comparedimmature myeloma cell lines with mature myeloma andnormal plasma cells, and reported that p16 expressionwas detected in the mature myeloma cells despite theabsence of cyclin D1. Cyclin D1 was the dominant proteinin the immature myeloma cells, whereas p16 was primarilyexpressed in normal plasma cells and mature myelomacells. According to the researchers, p16 amplification wasresponsible for the loss of long-term proliferation in someof the cell lines [49].

Although maximal expression of cyclin E occurredduring the G0/G1 phase in the present study, its expressiondid not differ significantly between the G0/G1 andthe S phases, the G0/G1 and the G2/M phases, or the S andG2/M phases. To date, cyclin E has not been observed inMM. Cyclin A expression was higher during both the S (P< 0.01) and G2/M (P < 0.05) phases than during the G0/G1 phase for MM group.

There wasn’t a significant difference in cyclin A expressionbetween the S and G2/M phases. Urashima et al.reported that p21 had widespread expression in MM celllines, independent of p53 [51]. They also reported thatp21 expression increased following exposure to dexamethasoneand downregulated by interleukin-6. Duringthe resting period (G1 arrest) expression of p21 was significantlyhigher, but decreased during proliferation. Similarly,we observed an increase in cyclin A expression and adecrease in p21 expression during the S phase, as comparedto the G0/G1 phase; however, in the control group cyclinA expression was significantly higher during the S phasethan during the G0/G1 phase, whereas p21 expression didnot differ. It can be an explanation to why proliferation isincreased when compared with the controls. Expression ofthe other mitotic cyclin—cyclin B—was similar to that ofcyclin A in the present study. Similarly, cyclin B expressionwas higher during the S (P < 0.05) and G2/M (P < 0.01)phases than during the G0/G1 phase, and there wasn’t asignificant difference between the S and G2/M phases. TheS phase fraction percentages in the CML and MM groups did not differ significantly than those in the control group.As expected, the highest S phase rate was observed in theCML group, followed by the control group, and the lowestrates were noted in the MM group, but there was no statisticallysignificant difference.

CML and MM present exhibit variable degrees of proliferation.It is noteworthy that in the present study we didnot isolate plasma cells in the MM patients and that thefindings reflect changes observed in bone marrow. Cyclinsand their inhibitors in the present study exhibited differentproperties during the cell cycle phases checkpoints in theCML and MM patients. Additionally, among the cyclins weexamined during different phases of the cell cycle, despitethe finding that some of them exceeded the normal range,cyclin inhibitors not associating this increase may contributethe mechanisms effective in the etiopathogenesis.

In conclusion, the present findings indicate that syntheticCDKIs may be a promising new treatment for CMLand MM.

## ACKNOWLEDGMENTS

This study was supported by the Scientific and TechnologicalResearch Council of Turkey (TUBITAK, Project no:SBAG-2205) and Turkish Academy of Sciences (TUBA).

## CONFLICT OF INTEREST STATEMENT

The authors of this paper have no conflicts of interest,including specific financial interests, relationships, and/or affiliations relevant to the subject matter or materialsincluded.

## Figures and Tables

**Table 1:**
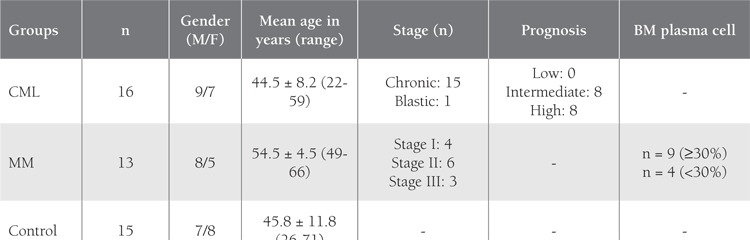
Characteristics of the Patients and Controls

**Table 2:**
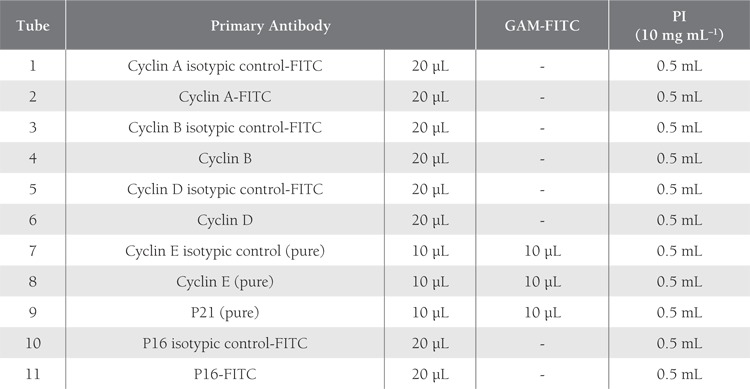
Pipetting Scheme

**Table 3:**
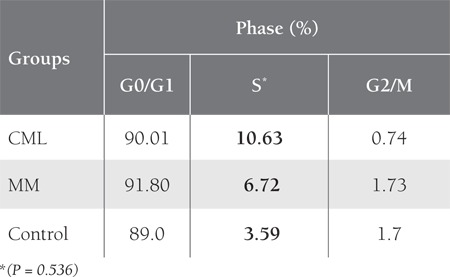
Cell cycle Phase Distribution in the Control and PatientGroups

**Table 4:**
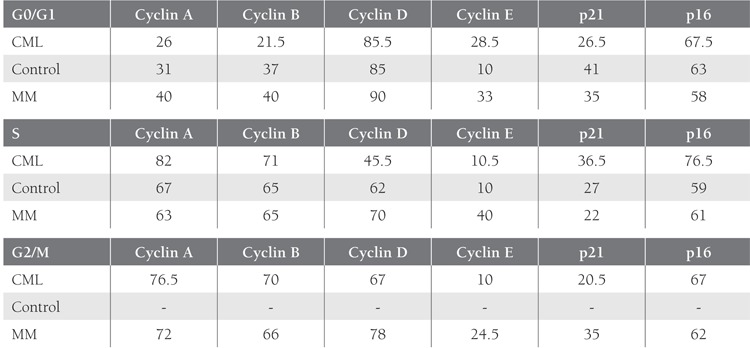
Percentage of Cells Expressing Cyclins and CDKIs, According to Phase

**Table 5:**
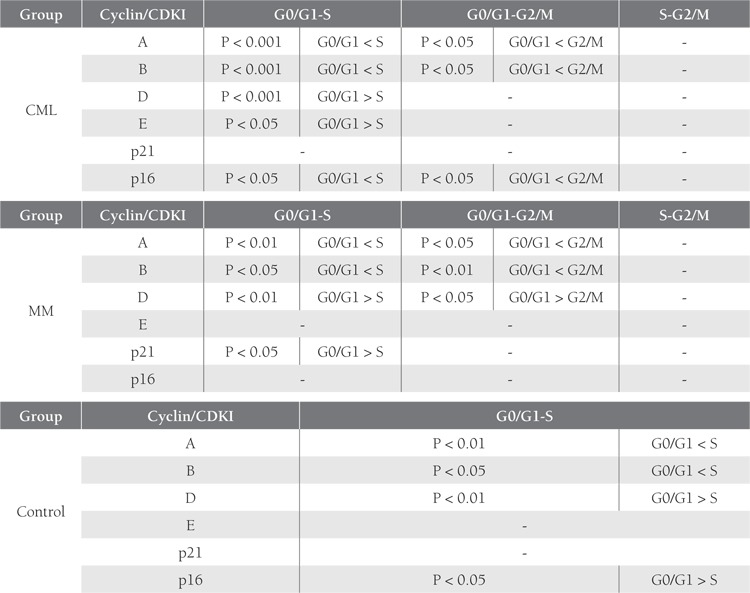
Comparison of Cyclins and CDKIs Between Phases in the Patient Groups

**Figure 1:**
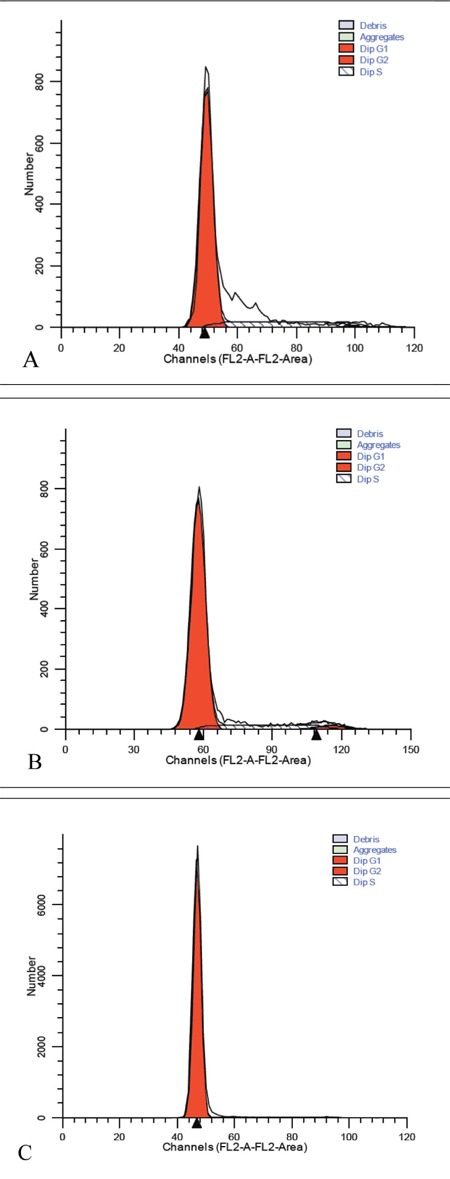
Distribution of the cell cycle phases analyzed usingModFit and CellQuest software (sample cases: A: CML;B: MM; C: control).
